# Consumer understanding of sugars claims on food and drink products

**DOI:** 10.1111/j.1467-3010.2012.01958.x

**Published:** 2012-05-11

**Authors:** N J Patterson, M J Sadler, J M Cooper

**Affiliations:** * Leatherhead Food ResearchLeatherhead, UK; † MJSR AssociatesAshford, UK; ‡ British Sugar PLCPeterborough, UK

**Keywords:** calories, consumer understanding, no added sugars, nutrition claims, reduced sugars

## Abstract

Consumer understanding of nutrition and health claims is a key aspect of current regulations in the European Union (EU). In view of this, qualitative and quantitative research techniques were used to investigate consumer awareness and understanding of product claims in the UK, focusing particularly on nutrition claims relating to sugars. Both research methods identified a good awareness of product claims. No added sugars claims were generally preferred to reduced sugars claims, and there was a general assumption that sweeteners and other ingredients would be added in place of sugars. However, there was little awareness of the level of sugar reduction and the associated calorie reduction in products when reduced sugars claims were made on pack. In focus groups, participants felt deceived if sugar reduction claims were being made without a significant reduction in calories. This was reinforced in the quantitative research which showed that respondents expected a similar and meaningful level of calorie reduction to the level of sugar reduction. The research also identified consumer confusion around the calorie content of different nutrients, including over-estimation of the calorie content of sugars. This is crucial to consumers' expectations as they clearly link sugar to calories and therefore expect a reduction in sugar content to deliver a reduction in calorie content.

## Introduction

The current paper focuses on nutrition claims used in food labelling, particularly sugar claims, such as ‘no added sugars’ and ‘reduced sugars’. Such claims highlight the beneficial nutritional properties of food and drink products by providing information about energy or nutrient composition, such as low fat or high fibre. They are a useful tool for communicating nutrition information to consumers and encouraging healthy eating patterns (van Trijp 2009; Richardson 2012) and are widely used by consumers (Food Standards Agency 2007; Grunert & Wills 2007).

In the European Union (EU), Regulation 1924/2006 (EU 2007) harmonises the use of nutrition (and health) claims to ensure effective functioning of the internal market and to provide a high level of consumer protection. Claims must not be false, ambiguous or misleading, or encourage excess consumption of a food. The presence, absence or reduced level of a nutrient or other substance for which a claim is made must have a beneficial nutritional or physiological effect, as established by generally accepted scientific evidence. The Regulation provides a list of permitted nutrition claims with conditions of use, including four claims relating to sugar content (see Table 1). A further claim is currently undergoing authorisation procedures.^1^

**Table 1:** List of permitted nutrition claims relating to sugar content (EU 2007)

Permitted nutrition claim	Conditions of use
Low sugars*	≤5 g sugars/100 g or ≤2.5 g sugars/100 ml
Sugar-free*	≤0.5 g sugars per 100 g or 100 ml
With no added sugars*	No added mono- or disaccharides or any other food used for its sweetening properties. If sugars are naturally present in the food, it should be labelled ‘Contains naturally occurring sugars’
Reduced (sugars)*	≥30% reduction compared with a similar product; the amount of energy in the product bearing the claim is equal to or less than the amount of energy in a similar product
Now contains *X*% less (sugars)*†	*X* is at least 15% compared with the product prior to reformulation, expressed per 100 g or 100 ml

* And any claim likely to have the same meaning for the consumer.

† Not yet authorised – undergoing scrutiny in the European Parliament.

Consumer understanding is a key aspect of the claims regulation, which states that ‘use of nutrition and health claims shall only be permitted if the average consumer can be expected to understand the beneficial effects as expressed in the claim’. A review of the current literature on consumer understanding of nutrition and health claims concluded that there is little clear-cut definitive research, that the evidence is often scant or conflicting and that less research has been conducted about nutrition claims compared with health claims (Food Standards Agency 2007). Hence, there is a need to expand the body of existing research on consumer understanding of claims to help underpin objective regulatory decisions (Food Standards Agency 2007; van Trijp 2009; Richardson 2012).

The present study investigated consumer understanding in the UK of reduced sugars and no added sugars nutrition claims, and consumers' perceptions of the calorie content of products bearing ‘reduced sugars’ claims. Qualitative and quantitative research methods were used to:

- assess awareness of general claims on food and drink products;
- understand consumers' associations with sugar and awareness of reduced sugars and no added sugars claims;
- assess the perceived calorie content of different dietary components and the perceived link between reduced sugars claims and calorie content;
- assess awareness of ingredients used to replace sugar.

## Methods

The research methods encompassed qualitative and quantitative consumer research techniques. Qualitative research (*e.g.* focus groups) was conducted with a relatively small number of participants and was designed to explore the breadth of consumer views. The results were used to inform follow-up quantitative research that assessed the frequencies of the responses in a wider population.

### Qualitative research

Four focus groups among main household shoppers were conducted by an experienced moderator in the qualitative research studio at Leatherhead Food Research, Surrey, UK, in July 2011, each lasting 60 minutes. The focus groups each comprised 9–10 participants (all female, all from Southern regions, with or without children and from a mix of social classes) who were recruited from Leatherhead's database. There were two groups with an age range of 20–45 years, and two groups with an age range of 46+ years. Exclusion criteria included diabetic subjects and those on restrictive diets, with food allergies or ‘food fads’. The structured discussion explored awareness of general claims on food and drink products followed by discussion of sugar, and awareness and understanding of reduced sugars and no added sugars claims. Discussion then focused on calories and participant understanding of the expected calorie reduction when reduced and no added sugars claims are used. Examples of products bearing such claims were then considered, followed by a final discussion on the future of sugar in the diet.

### Quantitative research

After analysis of the qualitative research findings, a questionnaire was constructed to enable quantification of the perceptions of claims on sugars identified by the qualitative research. The quantitative research was conducted via the Internet. Respondents from Leatherhead's consumer database were invited to participate, subject to the same exclusion criteria used for the qualitative research. Respondents were instructed to record their own thoughts and not to look up words or terms on the Internet. A total of 367 respondents completed the Internet survey (84% female, 16% male). Determined from the attitudinal questions used during the recruitment screening process, these were relatively ‘savvy’ consumers who claimed to regularly read food labels, with an age range of 18–70 years. The respondents were from households with 1 to >5 members, with 30% from households with at least one child. The questionnaire (see Appendix 1) was designed to quantify awareness of claims seen on food and drink products when shopping, as well as understanding of the calorie content of different nutrients, expected sugar reduction and associated calorie reduction in relation to ‘reduced sugars’ claims, and awareness of ingredients used to replace sugar in products.

## Results

### Qualitative research

During the initial discussion of claims seen on food and drink products, focus groups of both age ranges spontaneously recalled low fat claims. Younger age groups recalled wholegrain and energy claims, whereas older age groups recalled reduced and low cholesterol claims, and no added sugars and low sugar claims for children. A wide range of other claims were mentioned including some health claims (fuller for longer, locked up until lunch), country of origin claims (British produce), clean label claims (no artificial colours, no additives, no sweeteners), claims about food production methods (organic, free-range, quality standards) and other nutrition claims (high fibre, low salt). Claims that participants said they actively look for when shopping included low fat, low calorie, low salt, light and no sweeteners. However, not all participants actively looked for claims. Younger age groups were driven by products their children asked for, rather than by claims. They perceived claims to be hidden and that looking for them was too time consuming. In contrast, older age groups looked for a wider variety of claims. Participants' trust in claims ranged from a complete lack of trust, expressed by having to read all the ingredients and being ‘anti-claims’, through to complete trust, with a belief that claims have to be scientifically based and an assumption that claims are okay whichever manufacturer is making the claim (such that a brand association did not heighten belief in the claim). Younger age groups further expressed the view that claims can have a negative impact on purchasing decisions, mainly because products with low fat claims, for example, were expected to have less taste.

Participants' associations with sugar included sweetness, energy, nice taste, fattening, dental health, hyperactivity (younger age groups) and diabetes (older age groups). Beliefs about sugar included the belief that white sugar is refined and bad for you, brown sugar is more natural and healthier for you, sugar is okay in moderation, and that: ‘*The sugar in fruit is okay – but you can't have too much as that gets converted to fat*’.

When prompted with a reduced sugars claim, this was familiar to all participants and reactions were initially positive. Negativity was then expressed with regard to the expected taste, for example: ‘*I really don't like the taste, I'd rather have less of something I enjoy*’, and with regard to the replacement ingredients. Some participants were aware at this stage that the calories may not be that different because something extra is added in place of sugar. Participants spontaneously recalled a number of reduced sugars products and their beliefs about the reasons why sugar would be reduced were related to dental health, obesity (especially in children), diabetes and losing weight. When questioned about the level of sugar reduction, there was a lack of awareness of any guidelines, and participants suggested that it would be helpful for the level of reduction compared with the standard product to be stated on the pack. Expectations for sugar reduction ranged from 1% to 50%, and both older and younger groups settled on an ideal of 50% reduction. It was generally expected that artificial sweeteners would be added in place of sugars.

When prompted with a no added sugars claim, there was an assumption that the claim refers to sucrose rather than to total sugars. All participants expected there to be some form of sugars in a product labelled no added sugar/s, and all expected sweeteners to be added. Products with a claim for no added sugars were generally preferable to those with a reduced sugars claim as the process of not adding sugar was considered more ‘natural’ than taking something out (see Table 2).

**Table 2:** Statements from consumers in relation to ‘reduced sugars’ and ‘no added sugars’ claims (qualitative research)

Consumer statements regarding claims	

Reduced sugars	No added sugars
‘Some taken out’	‘Hopefully nothing done to it’
‘Something done to it’	‘Generally healthier than reduced sugars’
‘It's reduced in fructose, lactose and everything’	‘A different product altogether’
‘Not so good for you as no added sugars’	‘More natural than a product with a reduced claim’
‘Same product with less sugar’	‘Sounds better than reduced but when you get home you know you won't like it’

Some participants actively looked for calorie information on labels, particularly in the younger age groups and such information had been observed on many products. They selected some products on the basis of a ‘not too high’ calorie content (*e.g.* ready meals), but not treats or products for children. It was generally presumed that fat is the main nutrient taken out to reduce calories, sometimes sugar, and where calories are lowered there is a general expectation of less taste. Hence, some participants perceived that calorie claims are informative, whereas others found them to be off-putting. When asked about the associated calorie reduction for a 30% or 15% reduction in sugar compared with a standard product, the initial expectation was for the same level of calorie reduction, although following a discussion of calories, their expectations became revised downwards: ‘S*hould be the same (30%), that's your gut reaction, but if you think about it you realise it can't be … but when you're shopping you don't have time to think about it!’; ‘Should be similar reduction to the sugar … I've become quite down about all this, we buy these supposedly healthy things and they're just not’.* Participants felt deceived if sugar reduction claims were being made without a significant reduction in calories, and this was also seen as a frustrating revelation for those on a weight loss diet.

Drilling down further into consumer awareness of which nutrients have the most calories, in the qualitative research, alcohol followed by sugar was perceived to provide the most calories, followed by fat and carbohydrates: ‘s*ugar – because it turns to fat*’, ‘*carbs could have the most – it's the most filling so it would make sense*’. Protein was considered to have the least calories and to provide ‘good’ and ‘healthy’ calories. The perception was expressed that the calorie content of fat depends on the type of fat. Hence, there was general confusion regarding the calorie content of different macronutrients, with sugar being clearly associated with calories.

The participants were then presented with ten branded soft drinks to consider. There was a clear expectation that if the drinks were not labelled as ‘diet’, they would contain sugar and calories: ‘*All non-diet drinks must be sweetened with just sugar mustn't they?*’ and participants mostly recognised that the diet drinks would contain sweeteners. When it was revealed that many of the non-diet drinks contain a combination of both sugar and sweeteners, the participants were shocked and confused: *‘Are the sugar only ones lower in calories then?’; ‘I am amazed I didn't detect the sweeteners’*. It was also apparent that not all participants realised that ‘diet’ meant low calorie, reflecting a lack of understanding about the role of sweeteners.

Four food products were then discussed (baked beans, muesli, an instant hot beverage and confectionery) bearing different sugar claims (including reduced sugars and no added sugars claims) and with sugar reductions ranging from 29% to 49% compared with standard products. This prompted a general perception of less taste, an expectation of increased price and the realisation that some reduced sugars products contain less weight per similar size pack: ‘*This wouldn't fill me up – I'd need at least two!’*. There was shock at the low level of calorie reduction associated with the sugar reduction (1.4%–5.7%, with one product having increased calories per 100 g): ‘*There must be a high calorie value in whatever is replacing the salt and sugar to make it nearly the same’; ‘We would be suckers to buy the no added sugar version’.*

In the round-up discussion about the future of sugar, participants appreciated sugar as part of daily life and some recognised that although it is best in moderation, self-control to achieve this is challenging. All participants mentioned the obesity ‘crisis’ and how this has made consumers more health conscious. The older age groups considered that the public could be weaned off sugar in the future in much the same way as has occurred with salt (*i.e.* with a gradual reduction in the amount in products). Some participants thought there may be a link between sugar and hyperactivity: ‘S*ugar and sweets –“they” are all saying it causes hyperactivity’* and that knowledge of this (claimed) link would reduce the future consumption of sugar. The younger age groups were relatively cynical, hinting at a cycle of sugar being portrayed in the media as: *‘Good for you, then bad for you, then ok again … like eggs and butter’.* Some participants in the older age group felt they have little control over whether or not they have sugar in their diet: ‘*Manufacturers are being told they have to put in less sugar and salt, whether we actually want that is a different matter’.* Some participants in the younger age group wanted more transparency with the labelling of food products in the future: *‘Instead of trying to fool us, market all these things for what they really are’.*

### Quantitative research

Respondents were presented with a list of 14 main nutrition claims on food and drink products together with a ‘none of the above’ option and were asked which they had seen on food and drink products while shopping, which they actively looked for and which they sought for a child or grandchild (see Table 3). There was a broad awareness of these claims and respondents recognised an average of 12 out of the 14 claims. Eleven out of 14 claims had been seen by more than 80% of the respondents (Table 3). Nearly, all respondents (93.7%) had seen no added sugars claims and 81.7% had seen reduced sugars claims. Respondents stated that they actively looked for more than one-third of these claims. Almost half of the respondents did not shop for children or did not actively look for any of the claims while shopping for children. More than 3 out of 4 of those who actively looked for a claim when shopping for children cited they looked for no artificial colours; no added sugars was second, closely followed by no artificial sweeteners. Reduced sugars was the 6th most popular claim sought for children, with the top 6 all being negative claims (*i.e.*‘No …’, ‘Low …’ or ‘Reduced …’), as opposed to a positive addition to the product.

**Table 3:** Claims seen as well as actively sought while shopping (quantitative research)

		Claims actively looked for	
		
Claim	Claims seen (%)	For self (%)	For a child/grandchild (%)
No added sugars	93.7	52.0	40.1
Low fat	92.6	52.9	11.2
No artificial colours	91.6	45.2	43.6
Reduced fat	90.2	44.1	9.0
High in fibre	90.2	34.1	11.4
Light	89.9	31.3	3.3
Wholegrain	88.0	47.7	21.8
No artificial sweeteners	86.9	34.9	39.8
No preservatives	84.7	36.2	28.3
Reduced sugars	81.7	28.1	22.9
Low salt	81.7	36.0	29.7
Lowers cholesterol	75.5	20.2	1.9
Reduced calorie	65.4	22.1	3.0
Low GI	44.7	7.4	1.4
None of the above	1.1	9.5	43.9*

* Including ‘not applicable’ (*i.e.* do not shop for children).

Respondents were then asked to rank 8 dietary components in order of calorie content (see Appendix 1). In contrast to the qualitative research findings, saturated fat was ranked first by half of the respondents and fat was ranked first by over one-third of the respondents (Table 4). Hence, 85.6% of the respondents correctly ranked fat or saturated fat as having the highest calorie content. Nearly one-quarter of the respondents believed sugar to have the highest calorie content, and, on average, sugar was rated more calorific than alcohol and other carbohydrates. More respondents ranked salt as having the highest calorie content (9.7%) than carbohydrate (7.5%). This finding reflected the confusion apparent during the focus groups about the calorific content of various macronutrients and other dietary components.

**Table 4:** Ranking of perceived calorie content (quantitative research)

Mean score		1 %	2 %	3 %	4 %	5 %	6 %	7 %	8 %
**2.7**	Saturated fat	**49.9**	14.3	9.4	5.2	6.6	1.9	3.9	8.8
**2.8**	Fat	**35.7**	26.4	12.6	5.8	3.3	4.4	4.1	7.7
**3.3**	Sugar	**23.6**	14.4	23.6	11.7	9.7	8.6	3.3	5.0
**3.7**	Alcohol	11.6	17.7	**23.2**	17.7	11.9	8.6	5.5	3.9
**4.0**	Carbohydrates	7.5	11.1	17.2	**26.9**	22.4	9.4	3.0	2.5
**4.9**	Protein	2.5	5.9	17.0	14.4	15.9	**28.0**	10.5	5.7
**6.0**	Aspartame	5.2	6.4	5.8	8.7	4.9	10.2	**30.2**	28.5
**6.2**	Salt	9.7	5.7	2.3	4.0	6.3	4.5	22.2	**45.5**

There was also confusion about which nutrients or ingredients are important to watch out for with regard to weight control. Saturated fat was rated as the most important to watch, followed by calories and then by fat (Table 5). Overall, more than 4 out of 5 respondents considered either some type of fat or calories as most important to watch. Although sugar was perceived as relatively high in calories, only 7.4% considered watching sugar intake to be most important in order not to gain weight, suggesting good consumer awareness regarding messages about the link between high fat diets and weight control. In response to this question, respondents correctly identified that there is no energy in salt.

**Table 5:** Ranking of what is ‘important to watch’ if trying to avoid weight gain (quantitative research)

Prompted list	%
Saturated fat	31.9
Calories	27.2
Fat	22.1
Sugar	7.4
Carbohydrates	5.4
Alcohol	5.4
Protein	0.5
Salt	0.0

**Figure 1:**
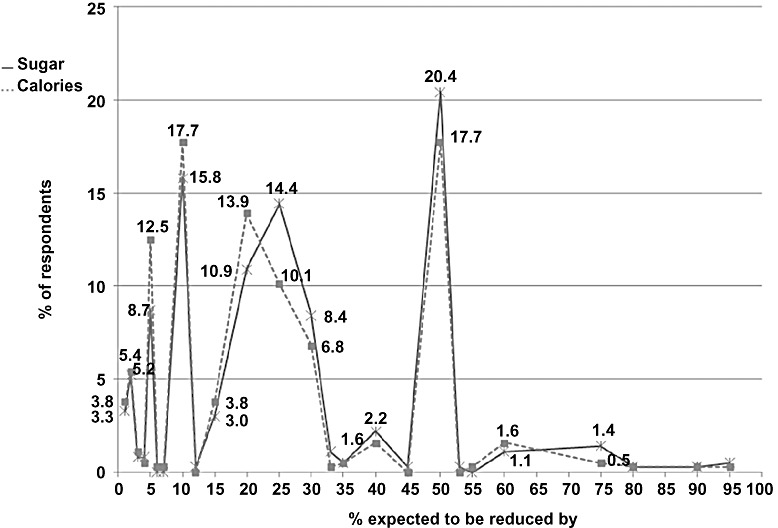
Expected sugar and calorie reduction in a reduced sugars product (quantitative research) (*n* = 367).

**Figure 2:**
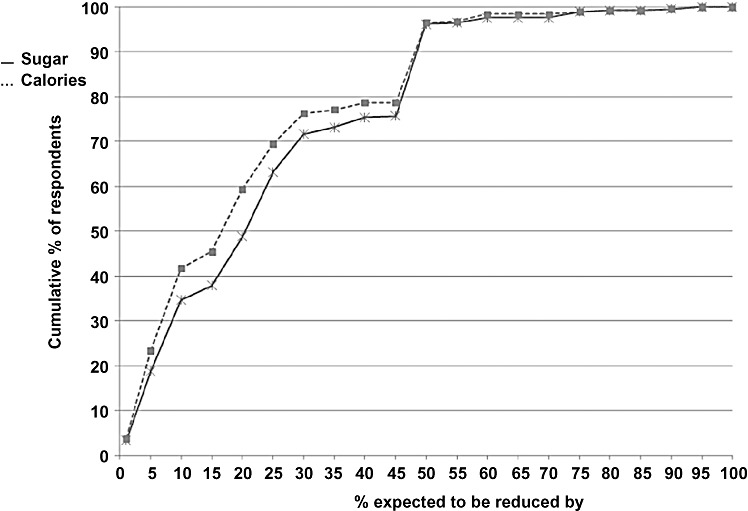
Expected cumulative sugar and calorie reduction in a reduced sugars product (quantitative research) (*n* = 367).

When questioned about the level of sugar reduction in food or drink products bearing reduced sugars claims, respondents clearly expected a similar and meaningful calorie reduction. More than half of the respondents expected a reduction of over 20%, whereas just over one-third expected only a 10% reduction or less. Very few respondents (less than 4%) expected a sugar reduction greater than 50%. The key finding was that respondents expected a similar level of calorie reduction to the level of sugar reduction, demonstrated by the cumulative expectations in calorie reduction being only marginally lower than a given level of sugar reduction (see Figs 1,2). That is, when 63.2% of the respondents expected reduced sugars to mean a sugar reduction of up to 25% compared with the original product, 69.5% expected an associated calorie reduction of up to 25%, and when 34.6% of the respondents expected a 10% sugar reduction, 41.7% of the respondents expected an associated calorie reduction of up to 10%.

Finally, respondents were presented with a prompted list of ingredients (sweeteners, saccharin, aspartame, fruit sugar, honey, xylitol, sugar, gum, stevia, fillers or ‘none of these’) and asked which they would expect to see in the ingredients of a no added sugars product and in a reduced sugars product. Respondents were almost four times more likely to expect products with a reduced sugars claim to contain sugars compared with products bearing a no added sugars claim. Reflecting participants' preference in the discussion groups for products with a no added sugars claim over a reduced sugars claim, the latter was expected to contain marginally more sweeteners, saccharin, aspartame, xylitol, gum, stevia and fillers than products with a no added sugars claim. Respondents were almost equally likely to expect fruit sugar or honey to be present in products with sugar-related claims. However, it was expected that products with no added sugars were more than twice as likely to contain none of the listed ingredients compared with reduced sugars products.

## Discussion

Consumer understanding of claims is one of the cornerstones of the nutrition and health claims regulation in Europe (EU 2007) and is thus of scientific, regulatory and food policy interest (Richardson 2012). The findings of the current research identify the need to further expand the body of research on consumer understanding in order to provide an objective base for regulatory decisions.

The current research focussing on consumer awareness and understanding of reduced sugars and no added sugars nutrition claims has highlighted high awareness of product claims of various types, including nutrition claims relating to sugars. There was a good understanding that reducing sugar means replacement with other ingredients, but there was poor awareness of the levels of sugar reduction achieved and a lack of awareness of the low level of associated calorie reduction in many food products. In the quantitative research, consumer expectations regarding the level of sugar reduction in reduced sugars products (more than half expected a reduction of over 20% and very few expected a reduction greater than 50%), suggest that the level of reduction required in the current legislation (30%) is in line with their expectations. In the qualitative research, participants were interested in being made aware of the actual level of sugar reduction compared with the standard product, which may help to increase consumer understanding. Claims for no added sugars were generally preferable to claims for reduced sugars, reflecting a preference for not adding something rather than taking something out.

The research has also highlighted consumer misconceptions about the calorie content of different nutrients and a lack of recognition that different types of fat and carbohydrates provide the same calories. This is crucial to consumers' expectations, as they clearly link sugar to calories and therefore expect a reduction in sugar content to deliver a reduction in calorie content, and they felt misled if this was not the case. With respect to which nutrients or ingredients it is important to watch out for in terms of weight control, consumers' low awareness of the high calorie content of alcohol highlighted in the quantitative research is noteworthy, particularly in relation to public health concerns.

Qualitative consumer research by the IGD has highlighted a number of areas of consumer misunderstanding about sugars, including that certain foods such as fruit and milk are rarely considered to be sources of sugar, and confusion about the best ways to consume sugars in a healthy balanced diet (IGD 2010). This is consistent with the current research that also highlighted a number of areas of consumer confusion including perceived differences in the extent of refining between white and brown sugars, overestimation of the amount of calories in sugar and misconceptions about commonly held associations with adverse health effects (*e.g.* diabetes and hyperactivity).

A review of consumer understanding of claims made on food identified that consumers like short adjectival descriptors such as ‘low’, ‘free’ and ‘no added’ in nutrition claims as they are readily understandable and allow consumers to make quick comparisons between products (Food Standards Agency 2007). However, the review also highlighted that consumers can be misled by some types of simple information. This is borne out by a study that identified the potential for low fat nutrition claims to provide a ‘health halo’ on products bearing such claims and suggested that this effect may occur with other relative nutrition claims such as ‘reduced calorie’ (Wansink & Chandon 2006). It is also apparent from the current research that consumers feel misled by a small calorie reduction in a reduced sugars product as they clearly expect a reduced sugars claim to be associated with a meaningful calorie reduction that is similar to the level of sugar reduction. If mono- and disaccharides (*e.g.* sucrose or glucose) providing 4 kcal/g are reduced and replaced with non-nutritive sweeteners, this can result in a significant calorie reduction, and this can be achieved in products where sugar is the main energy source and where its main function is to impart sweetness (*e.g.* sugar-sweetened beverages). However, in more complex food matrices where sugars contribute a number of technological functions, any calorie reduction is dependent on the ingredients used to replace the sugars and, hence, calorie reductions are often limited. If, for example, a higher proportion of fat results from replacing sugar in a product, the calorie content per unit weight will increase. The current research has identified that consumers are clearly unaware of this, and expect sugar reduction to be associated with a meaningful calorie reduction.

The Food Standards Agency review also highlighted conflicting reports about whether consumers understand that no added sugars could mean that the product contains naturally occurring sugars (Food Standards Agency 2007). In the current research, participants expected that products with no added sugars claims would contain some form of sugar such as fructose, honey and other naturally occurring sugars.

In conclusion, this research has identified that consumers expect reduced sugars claims to be associated with a similar and meaningful level of calorie reduction and feel misled if this is not the case. The research has also highlighted a high level of consumer confusion regarding the calorie content of different macronutrients. Overall, these conclusions highlight many important aspects of communicating nutrition messages to consumers and the potential for some claims to confuse and mislead.

## Footnotes

1 Subsequent to submission of the manuscript this claim was rejected by the European Parliament on the grounds that it would mislead and confuse consumers.

## Appendix 1

### Quantitative questionnaire

Think about each of the following questions carefully before responding. Please do not look up any of the words/terms on Google, we just want to know what you think when you see the word, not what someone else thinks. The information will be useless to us if everyone has text book answers; we value your honest thoughts and opinions. Thanks in advance, Leatherhead Consumer Team.

## References

[b1] EU (European Union) (2007).

[b2] Food Standards Agency (2007).

[b3] Grunert KG, Wills JM (2007). A review of European research on consumer response to nutrition information on food labels. Journal of Public Health.

[b4] IGD (2010).

[b5] Richardson DP (2012). Preparing dossiers: strength of the evidence and problems of proof. The Proceedings of the Nutrition Society.

[b6] van Trijp HC (2009). Consumer understanding and nutritional communication: key issues in the context of the new EU legislation. European Journal of Nutrition.

[b7] Wansink B, Chandon P (2006). Can low fat nutrition labels lead to obesity?. Journal of Marketing Research.

